# The life cycle of SPβ and related phages

**DOI:** 10.1007/s00705-021-05116-9

**Published:** 2021-06-07

**Authors:** Katharina Kohm, Robert Hertel

**Affiliations:** FG Synthetic Microbiology, Institute for Biotechnology, BTU Cottbus-Senftenberg, 01968 Senftenberg, Germany

## Abstract

Phages are viruses of bacteria and are the smallest and most common biological entities in the environment. They can reproduce immediately after infection or integrate as a prophage into their host genome. SPβ is a prophage of the Gram-positive model organism *Bacillus subtilis* 168, and it has been known for more than 50 years. It is sensitive to dsDNA damage and is induced through exposure to mitomycin C or UV radiation. When induced from the prophage, SPβ requires 90 min to produce and release about 30 virions. Genomes of sequenced related strains range between 128 and 140 kb, and particle-packed dsDNA exhibits terminal redundancy. Formed particles are of the *Siphoviridae* morphotype. Related isolates are known to infect other *B.* *subtilis* clade members. When infecting a new host, SPβ presumably follows a two-step strategy, adsorbing primarily to teichoic acid and secondarily to a yet unknown factor. Once in the host, SPβ-related phages pass through complex lysis–lysogeny decisions and either enter a lytic cycle or integrate as a dormant prophage. As prophages, SPβ-related phages integrate at the host chromosome's replication terminus, and frequently into the *spsM* or *kamA* gene. As a prophage, it imparts additional properties to its host via phage-encoded proteins. The most notable of these functional proteins is sublancin 168, which is used as a molecular weapon by the host and ensures prophage maintenance. In this review, we summarise the existing knowledge about the biology of the phage regarding its life cycle and discuss its potential as a research object.

## Introduction

**Fig. 1:**
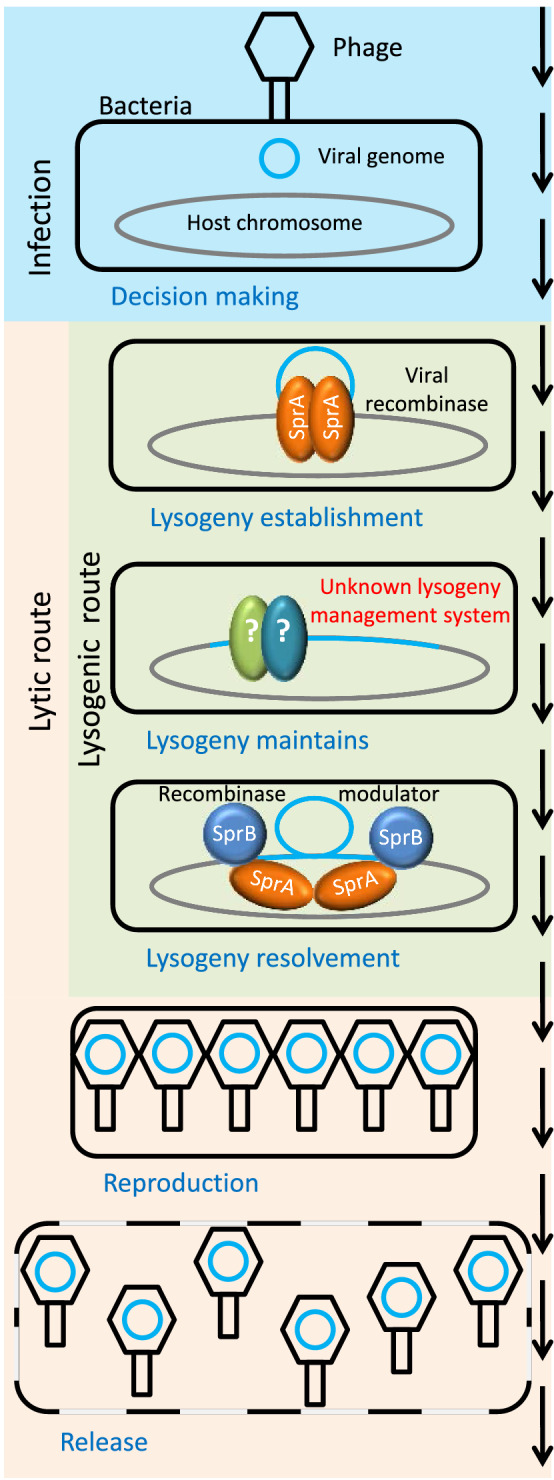
The life cycle of SPβ and related phages

Phages are viruses of bacteria and are the smallest and most common biological entities in the environment. As viruses, they depend on the metabolism of their bacterial hosts for reproduction. During the reproductive process, most phage types completely consume the resources of their hosts and kill them when releasing their progeny [1, 2]. Phages that reproduce immediately after infection are called lytic phages (lytic life cycle). However, some phage types are able to reproduce *via* a temperate life cycle (Fig. 1) in which they insert their genetic information into the genome of the host bacterium, thus becoming prophages, which subsequently multiply passively through the growth of their host. The process of prophage incorporation into the host chromosome is called lysogenisation, and the resulting bacterium with the prophage is called a lysogen. The genetic material of the prophage is transferred to the daughter cells with each cell division. The bacterium can proliferate without any disadvantages (apart from increased energy expenditure for prophage DNA replication), together with its inactive prophage. Sometimes the lysogen acquires a competitive advantage through the presence of its prophage in the form of phage resistance [3, 4] or prototrophy [5, 6]. In rare cases, a prophage can even turn the bacterium into a pathogen (lysogenic conversion). For example, the pathogenicity of **e**ntero**h**emorrhagic ***E****scherichia ***c***oli* (EHEC) and *Vibrio cholerae* is due to the presence of a prophage [7, 8]*.* When the bacterial cell is exposed to stress, the prophage can be activated and enter the lytic life cycle again.

SPβ infects the Gram-positive model bacterium *Bacillus subtilis* 168 and, as a prophage, significantly impacts the properties of its host. Like its host, it has great potential to serve as a model system for phage biology. In this review, we summarise the existing knowledge about the life cycle of phage SPβ and its impact on its host bacterium.

## Host system

*Bacillus subtilis* was first described by W. Cohn in 1875 as a small sporulating bacterium. The original strain was lost, and in 1930, H. J. Conn proposed the Marburg strain as the new type strain because it best matched the original descriptions of *B.* *subtilis* [9], which was soon accepted by the scientific community [10]. In 1947, Burkholder and Giles exposed this strain to a sublethal dose of X-rays and generated a tryptophan-auxotrophic strain named 168 [11]. This strain was distributed worldwide due to its high transformability, as shown by John Spizizen [12]. Thus, *B.* *subtilis* 168 became a model organism for many aspects of bacterial molecular biology [13] and one of the most frequently used hosts for *B.* *subtilis* phages [10].

The genome sequence of *B.* *subtilis* 168 was first published in 1997 by Kunst et al. [14], was resequenced by Barbe et al. in 2009 [15], and has since undergone frequent annotation updates [16, 17], making it one of the best-characterised bacterial genomes. The current version of the genome sequence indicates that it consists of one chromosome 4,215,606 bp in size with 34.98% GC content. It contains 4,325 protein coding regions and encodes 86 tRNAs, 30 rRNAs, two ncRNAs, and 93 small RNAs (misc_RNA). Furthermore, it harbours one integrative and conjugative element (ICEBs1) [18], four prophage-like-regions, and two prophages, known as PBSX [19] and SPβ [20]. All of these alien genomic elements are non-essential for the lysogen and can be removed from the genome of *B.* *subtilis* 168 [21].

*Bacillus pumilus*, *Bacillus licheniformis*, and *Bacillus amyloliquefaciens* are other well-known representatives of the *B.* *subtilis* clade [22, 23]. These species are morphologically very similar and are mainly mesophiles and neutrophiles [23]. Members of this clade are frequently suitable hosts for phages that were initially isolated on *B.* *subtilis*, such as φ29 [24, 25], SP-15 [26], or SPO1 [27], and for diverse SPβ-related phages, as recent bioinformatic analysis has demonstrated [28].

## SPβ and related isolates

**Fig. 2:**
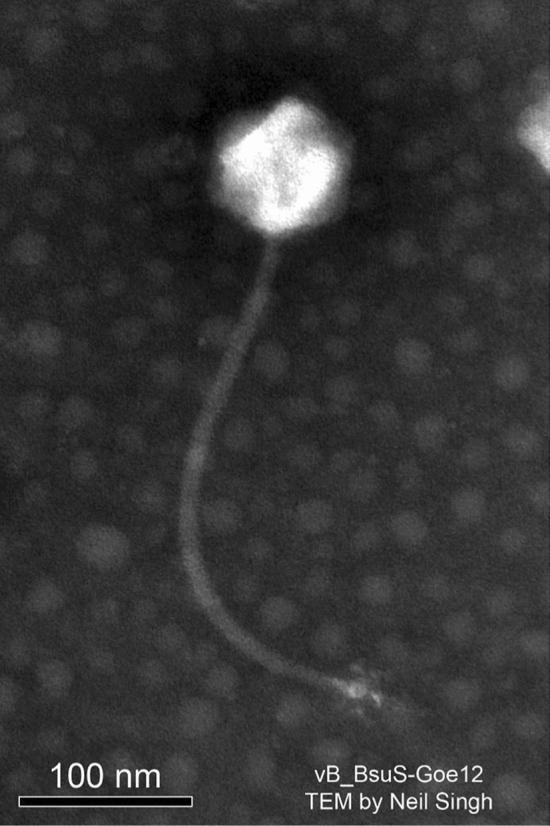
Virion of the SPβ-like phage Goe12 (vB_BsuS-Goe12)

The characterisation of SPβ was first described in the PhD thesis of F. A. Eiserling at the University of California at Los Angeles in 1964. Two "defective" phages of *B.* *subtilis* 168 were investigated and named SPα and SPβ [10]. Edna Seaman and co-workers independently characterised both phages a second time and named them PBSX (SPα) and PBSY (SPβ) [19]. However, the designation SPβ has become generally accepted. SPβ has an icosahedral head (82 to 88 nm in diameter) and a 12-nm-wide and 320-nm-long flexible non-contractile tail, with a 36-nm-wide baseplate exhibiting six equidistant, radial projections [10, 29]. Thus, it resembles the *Siphoviridae* morphotype (Fig. 2), like the small temperate *B.* *subtilis* phage φ105 [30] and the small lytic phage SPP1 [31].

This phage was not further investigated until the discovery of *B.* *subtilis* CU1050, a strain that was spontaneously cured of SPβ and is therefore suitable for its lytic replication [29, 32]. Many SPβ-related phage isolates have been reported in the literature, including IG1, IG3, IG4 [33], φ3T [34], Z [33], ρ11 [35], and SPR [36]. Almost all are directly associated with *B.* *subtilis*. IG4 can also be propagated on *B.* *pumilus* [33], and although H2 originated from a *B.* *amyloliquefaciens* lysogen [37], it can also lysogenise *B.* *subtilis* [38].

**Fig. 3:**
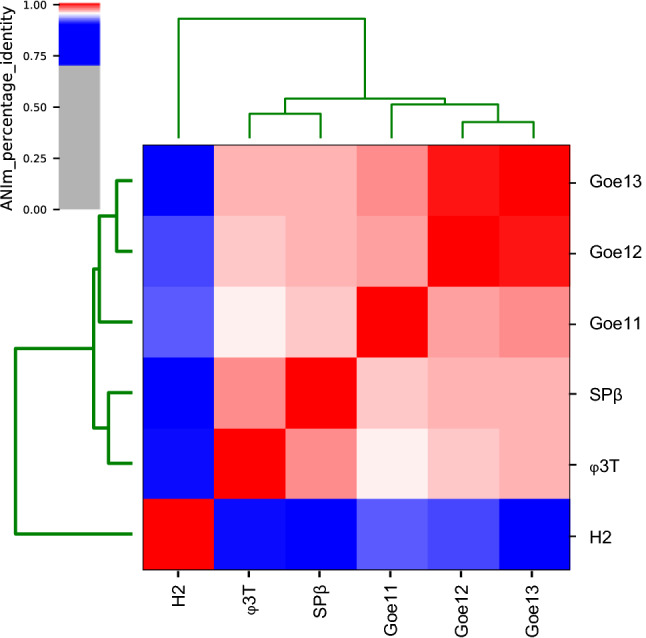
Average nucleotide sequence identity of SPβ-related phages. Blue–white (70–95% identity) indicates affiliation to the same genus; and white–red (95–100% identity), to the same species.

SPβ-related phages fall into three subgroups [39, 40]. Phages IG1, IG3, IG4, ρ11, and φ3T are particularly closely related to SPβ, as SPβ antiserum cross-reacts with all of these phages [33, 39]. SPR belongs to the second subgroup. Antiserum against this phage does not inactivate the above-mentioned strains. In addition, the SPR viral DNA exhibits a unique methylation pattern [36]. Phage H2 represents a third subgroup [37, 38]. It has less genome sequence similarity to other SPβ-like phages and a specific antiserum activity [39]. Three recent isolates, Goe11 [MT601272.1], Goe12 [MT601273.1], and Goe13 [MT601274.1], have been sequenced. Average nucleotide sequence identity analysis shows them to belong to the same species as SPβ and to cluster together with SPβ and φ3T (Fig. 3).

## SPβ induction and release

The availability of a suitable host strain has enabled the investigation of SPβ in more detail and and verified it as functional and capable of lytic reproduction. It forms small turbid plaques on the SPβ-free strain CU1050 [29, 32]. As prophage, it is activated by mitomycin C and thus reacts to DNA damage [29]. If induced in *B.* *subtilis* 168, it has a latent period of about 90 min and a burst size of 28-36 viable particles. The clear-plaque mutant SPβ c1 is incapable of lysogenising its host. It has a shorter reproduction cycle of only 46 min and a reduced burst size of 16 progeny particles [29]. However, as this phage mutant was never sequenced, the genetic basis of its accelerated reproduction remains unclear.

When mitomycin C enters into bacterial cells, it intercalates into the dsDNA, forming cross-links that in turn cause stalling of the replication fork and RecA activation [41–43]. Activated RecA stimulates proteolytic auto-cleavage of (i) the LexA repressor, thereby activating the SOS response, and (ii) phage repressors, thereby leading to prophage induction, as has been proposed as an induction mechanism for the PBSX prophage [37]. SPβ induction by mitomycin C most likely relies on a more complex mechanism. The YonR protein of SPβ shows pronounced similarity to the lysogenic repressor Xre for lysogeny maintenance of PBSX and YqaE of the *skin* element, a degenerated prophage of *B. subtilis* 168. YonR has been proposed to be the lysogenic repressor of SPβ [44]. The d protein (YomJ) was identified as a further lysogeny management component. If expressed ectopically from a plasmid, it conveys resistance against closely related phage strains [45]. However, neither *yonR* nor *yomJ* seems to be essential for maintaining the lysogenic state of SPβ, as both could be individually deleted from the prophage genome [46]. These facts suggest a lysogeny control mechanism involving two independent repressors and/or host components, or an activator-based switch to the lytic pathway. Each of the proposed cases would present a new system that is worth scientific attention.

There are indications of the involvement of a host-specific component in SPβ induction. A genomic analysis of SPβ by Lazarevic et al. revealed the presence of four SOS boxes, two in front of the divergently transcribed genes *yolC* <-> *yolD*, one at *yopS* <-> *yopT*, and one in front of *yorB* [44]. Further analysis by Au et al. led to the identification of three additional SOS boxes associated with SPβ-related genes (Table 1) [47]. Those SOS boxes provide a binding site for LexA, also known as DinR (damage-inducible regulator) [48]. Au and co-workers were able to confirm *in vitro* binding of LexA to the promoter regions of the *yorB*, *yolC*, and *yolD* genes. However, it remains unclear how the SOS-box-associated genes of SPβ respond to SOS induction in the host. For technical reasons, *B. subtilis* YB886, a genetic descendant of the SPβ-free strain CU1050 [29, 32, 49], was used for those experiments. LexA is not responsible for silencing SPβ, as it can be deleted from the host genome without activating the prophage [46]. The possibility that a combination of host- and phage-derived components such as LexA and YonR may handle this task jointly remains to be explored. Another possible host factor involved in SPβ maintenance is the extracytoplasmic function (ECF) sigma factor SigY [50]. Deletion mutants of SigY spontaneously lose the SPβ prophage.

**Table 1:** SOS boxes identified in the SPβ genome

Gene^a^	SOS box^b^	Position^c^	*K*_d_^D^ (nM)	No. of mismatches
*yokF*	a**GAAC**AaAc**aTTC**t	− 17	n. d.	5 (1)
*yolC* (1)	a**GAAC**AaAc**GTTC**t*^1^	− 127 (− 74)	3.9	4 (0)
*yolC* (2)	a**GAAC**AaAa**GTTC**G*^2^	− 97 (− 44)		3 (0)
*yolD* (1)	C**GAAC**tTtT**GTTC**t*^2^	− 64 (− 17)	3.9	3 (0)
*yolD* (2)	a**GAAC**gTtT**GTTC**t*^1^	− 34 (+ 14)		4 (0)
*yonT*	C**GAAC**ATAa**GTTt**t	− 320	n. d.	3 (1)
*yopS*	g**GAAC**gTgc**GTTC**t*^3^	− 119	n. d.	5 (0)
*yopT*	a**GAAC**gcAc**GTTC**c*^3^	− 51	n. d.	5 (0)
*yorB*	a**GAAC**ActT**GTTC**c	− 62	12	4 (0)
*yorL*	a**GAAC**tTgT**GTTt**t	− 15	n. d.	5 (1)
Consensus	C**GAAC**ATAT**GTTC**G			

The data originate from reference [47]

*The operator instance to regulate different genes on the leading and lagging strands

^a^Genes are listed by the position in the SPβ c2 genome. The numbers in parentheses indicate different operators within the same promoter region

^b^Lowercase nucleotides and uppercase nucleotides indicate nonconsensus and consensus residues, respectively. The underlined sequences were identified previously by Lazarevic et al. [44]

^c^Location of the 3' end of the SOS box relative to the ATG codon of the respective gene (and relative to the 3' end of the -10 region of the canonical SigA promoter sequence)

^d^Apparent binding constant of LexA determined by Au et al. [47]

Once SPβ is induced, SprA and SprB proteins manage its excision. The recombination directionality factor SprB promotes synapsis of SprA subunits bound to the *attL* and *attR* sites and releases the phage genome with the simultaneous reconstitution of the *spsM* gene [51, 52]. While the *sprA* gene is constitutively expressed [53, 54], the expression of the *sprB* gene depends on the activity of the SigK or SigE sigma factor [52] or a stress-induced SigA promoter [53]. These different promoters enable the excision of the SPβ genome, not only upon induction with mitomycin C (SigA) but also during sporulation (SigK and SigE), where SPβ is removed from the genome to re-establish the SpsM function and thus ensure proper spore surface glycosylation (see below) [53]. A similar prophage excision and gene re-establishment during sporulation was observed with the *kamA* gene when disrupted by the φ3T prophage [55].

**Fig. 4:**
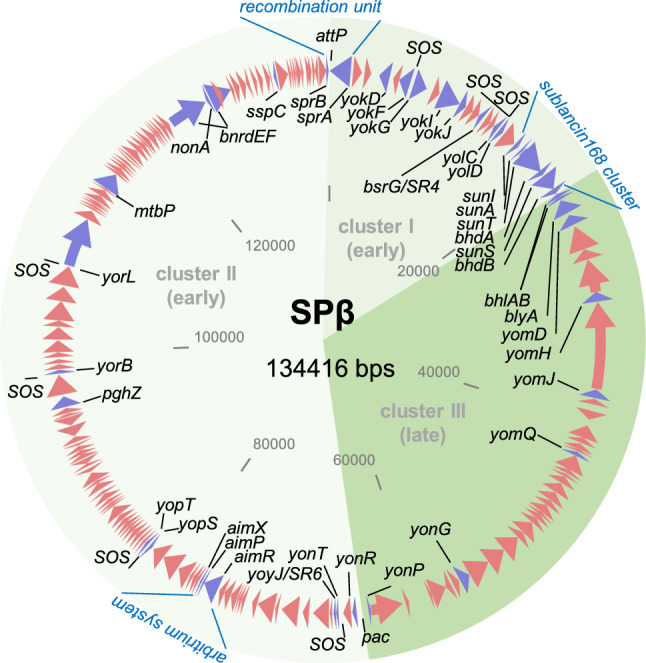
The genome of *Bacillus* phage SPβ. The genome orientation and the locations of its genomic clusters I–III are defined with respect to phage replication (I, early; II, early; III-late) and adjusted according to Lazarevic et al. [44]. Arrows indicate protein-coding genes. Red arrows represent genes encoding hypothetical proteins not discussed in this review. Purple arrows represent genes discussed in this review to which gene names are connected with a black line. The *attP* site, the *pac* site, the SOS boxes, the recombination unit, the sublancin 168 cluster, and the arbitrium system are indicated. The genome map's initial structure was created with Clone Manager 8 (Sci Ed Software, Westminster, Colorado, USA) using the SPβ c2 genome sequence [44] and elaborated further with MS PowerPoint 2019.

So far, members of the SPβ group are the largest known temperate phages of *B.* *subtilis*. Their genomes range in size from 128 and 140 kb (SPβ c2, 134 kb [44]; φ3T, 128 kb [56]; H2, 140 kb [positions 1979051..2118711 in CP041693]). Fig. 4 shows a circular map of the SPβ phage genome that is representative of the entire phage group. In the *B.* *subtilis* 168 chromosome, SPβ is integrated into the *spsM* gene, creating two truncated genes: *yodU* (BSU_19810) and *ypqP* (BSU_21670). When excised from the bacterial chromosome during sporulation or phage induction, the *spsM* gene is reconstituted. It codes for a sugar epimerase, which is needed for spore polysaccharide coating by the mother cell during sporulation [53]. The *sprA* (BSU_21660) and *sprB* (BSU_19820) genes are essential for the excision process. Both are located at the outer margins of the SPβ prophage and thus mark its boundaries. In the circular SPβ genome, these genes flank the *attP* site of the phage [53].

Upon induction, SprB modulates SprA to excise the SPβ genome from the host chromosome and presumably circularise it for replication [52, 53]. With the *bnrdEF* genes, SPβ provides an additional ribonucleotide reductase, likely to meet the increased demand for deoxyribonucleotides during its replication. Apart from the significant similarity of the phage-encoded system to the host system (*nrdEF*), each of the phage ribonucleotide reductase genes contains an intron, and *bnrdE* harbours an additional intein-coding sequence. Whether these mobile elements fulfil a function during phage replication or are dispensable remains unclear [57, 58].

The replication processes of SPβ have not yet been investigated. It can just be speculated to be similar to that of SPP1, which is capable of theta and sigma replication (reviewed in reference [59]). Sigma replication, also called rolling-circle replication, results in a phage genome concatemer, which serves as a substrate for genome packaging. Before genome packaging, MtbP of SPβ methylates the first cytosine of the GGCC palindrome at position 5 of the pyrimidine ring [36, 44, 60, 61]. In this way, the SPβ genome is protected from the host's defence systems during the next infection cycle [62].

The genome packaging mechanism of SPβ also remains to be elucidated, but it is also likely to be similar to the one used by SPP1. Different SPβ mutants (SPβ c1 del1, SPβ c2 del2, SPβ c2 del3, and SPβ c2 del4) have been constructed with an about 10% reduced genome size relative to that of the wild type [63, 64]. When a *cos* site is used for phage genome maturation, the genome is cut into defined units during packaging [65], but in the case of these deletion mutants, the phage head is not filled correctly with DNA due to reduced genome size, resulting in unstable viral particles with reduced viability [66]. However, to our knowledge, such observations have not been reported for SPβ mutants bearing deletions [64]. Alternatively, encapsidation can be initiated at a *pac* site, employing a head-full mechanism [67]. In this case, the *pac* site is cleaved, and the phage genome's concatemeric DNA is unidirectionally translocated into the interior of a procapsid until the phage head is full. A sequence-independent DNA cut terminates the process. The encapsidated genome exceeds 100% and thus contains terminal redundancy, which is later necessary for re-circularisation. With phage SPP1, when the first head is filled following the first concatemer cut, the remaining dsDNA is used directly for further encapsidation cycles. As a result, a heterogeneous population of terminally redundant and partially circularly permuted DNA molecules is generated (reviewed in reference 68). Thus, a shortened SPβ genome would lead to elongated terminal redundancies while still forming a correctly filled head and stable particles. Sequence read distribution analysis of new SPβ-like isolates (Goe11, Goe12, and Goe13) strengthens the assumption of a *pac* site and headful-based genome packaging by SPβ-like phages (our own unpublished data).

Restriction mapping studies of SPβ from particle-packed genomic DNA have indicated the location of the potential *pac* sites between or around *yonR* (BSU_21020) and *yonP* (BSU_21030) [69]. However, a comparison with the SPP1 *pac* site [70] revealed no sequence similarities (data not shown).

## Virion assembly and release

The SPβ gene cluster between *yonP* and *bhlB* has been proposed to contain structural genes required for particle assembly [44]. This cluster is transcribed as a single polycistronic mRNA by the SPβ-specific single-subunit RNA polymerase YonO, which is active at the late stage of replication [71].

Almost nothing is known about the virion assembly of the SPβ phage. The *yomH* gene, annotated as "tail family protein" [NC_001884], and the *yomQ* gene, annotated as "putative tail phage assembly protein" [NC_000964], are the only two genes where annotation indicates a potential involvement in particle formation. Even an automatic annotation of the recently sequenced genome of a *B.* *subtilis* 168 derivative [72] includes no new information about structural genes of SPβ.

The gene cluster from *yomD* to *bhlB* (nt 2,262,437-2,265,169 in NC_000964) can be associated with cell lysis required for the release of viral progeny. The *yomD* gene (BSU_21400) codes for a hypothetical protein, which shows no homology to any known domain or structure (InterProScan 20200716, data not shown). It overlaps with a gene (BSU_21409) that is separated from *blyA* (*yomC*) by an 18-bp-long intergenic region. The *blyA* gene codes for *N*-acetylmuramoyl-L-alanine amidase, which resembles the phage lysin [73]. The proximity of these three genes implies a joint function, which awaits experimental confirmation. The other two genes of this cluster, *bhlA* (*yomB*) and *bhlB* (*yomA*)*,* code for potential holin-like proteins, which presumably initiate membrane permeabilisation required for the phage lysin to reach its target and lyse the host cell [73].

## Infection and Immunity

Finally released by bursting the host cell, SPβ may start a new infection cycle. This might be challenging, as a *B.* *subtilis* population often forms an extracellular matrix consisting of poly-γ-glutamic acid (γ-PGA), which can prevent the phage from gaining access to the host cell [74]. However, the virulent *B.* *subtilis* phage phiNIT1 can overcome this barrier by employing a phage-derived γ-PGA hydrolase [74]. The SPβ-derived gene *pghZ* (BSU_20460) codes for such a functional γ-PGA hydrolase [75], and this might help SPβ to overcome the γ-PGA barrier.

Like some other *B.* *subtilis* phages, SPβ primarily adsorbs to the teichoic acid of the host cell wall [40, 76]. The possibility that it uses a protein as a second receptor protein, like the morphologically related lytic phage SPP1 [77], remains open. Phage SPP1 adsorption occurs in two steps, the first of which is reversible but more efficient. The second step is irreversible and associated with the cell wall protein YueB, but it is unlikely to happen without prior reversible association with cell-wall teichoic acid [78]. Without the correct teichoic acid, SPP1 can still bind to YueB, but with reduced efficiency and preferably on solid media [78]. SPβ may follow a similar adsorption strategy. *B.* *subtilis* strain IGCgll4, an SPβ-resistant strain, has been reported to harbour a mutation affecting the biosynthesis of teichoic acid. This mutation abolishes the adsorption of SPβ and confers resistance to this phage [40]. However, the observed resistant phenotype may be a misinterpretation. Reduced adsorption strongly impacts plaque formation and lysis of the host culture, mainly because the surrounding host cells grow faster than the phages can infect them [79]. This assumption is supported by transduction experiments showing the ability of SPβ to inject DNA into strain IGCgll4 [39]. These results imply a secondary attachment site for SPβ to which the phage adsorbs poorly.

## Once in the host, which lifestyle to choose?

Besides a direct lytic replication a direct lytic replication cycle that kills the host, SPβ, as a temperate phage, can also coexist with the host by becoming a prophage via integration of its DNA into the host chromosome, thereby generating a lysogenic bacterial strain. In its prophage form, SPβ propagates passively through the replication of its host [80].

The choice between a lysogenic and a lytic lifestyle was investigated for φ3T, a close relative of SPβ [56]. The "arbitrium" system of this group of phages is the first step in the decision process. It relies on small-molecule communication to execute lysis–lysogeny decisions [56]. The arbitrium system consists of AimR, a transcription activator; AimP, a quorum-sensing signal peptide; and AimX, a non-coding RNA (ncRNA), which is a lysogeny activator or lysis repressor. AimP is a small peptide with an N-terminal signal sequence that allows it to be secreted into the medium via the *B. subtilis* Sec pathway. Outside the cell, it is further processed by extracellular proteases to generate the final hexapeptide, which is reimported by the host oligopeptide transporter (OppABCDF) into the cytosol, where it interacts with the AimR regulator. The AimR regulator, when free of AimP, binds as a homodimer to the *aimX* operator, represented by two hexameric inverted repeats separated by 25 bp [81], and promotes *aimX* transcription (lytic cycle). The φ3T phage AimR dimer dissociates to monomers after interaction with the AimP peptide, the concentration of which increases during infection of a host population. The monomeric AimR cannot bind its operator and promote *aimX* transcription [56], thus favouring lysogeny. In SPβ, the association of the AimR dimer with the AimP peptides leads to a closed conformation of the AimR dimer that makes it unable to bind its operator [81] and promote the expression of the *aimX* ncRNA gene. Crosstalk of the arbitrium systems of different phage strains may be feasible, but it has already been found that φ3T and SPβ have incompatible systems, even though they belong to the same subgroup [56, 81].

Recent findings indicate that the integrative and conjugative elements ICE*Bs1* may take the choice of lysis–lysogeny from SPβ. The SpbK protein, encoded by the *spbK* gene on ICE*Bs1*, interferes with the lytic reproduction of SPβ and thus leaves this phage only the option of lysogenisation [82]. This action implies an attempt by the host and its ICE*Bs1* element to "domesticate" SPβ.

## Establishing lysogeny

When establishing lysogeny, a long-term relationship between the host bacterium and the phage in its prophage form, SPβ-related phages preferably integrate at the replication terminus of the bacterial chromosome [5, 28, 83, 84]. The phage attachment site sequence (*attP*) for SPβ has been identified [52]. On the circularised phage genome, it is located between the *sprA* (BSU_21660) and *sprB* (BSU_19820) genes, has an AT-rich core region, and is flanked by inverted repeats. The corresponding bacterial attachment site sequence (*attB*) is located in *spsM*, which is interrupted upon integration, resulting in two pseudogenes: *ypqP* and *yodU* [53].

Regarding historical SPβ-related strains, the genome sequence is available only for φ3T and H2. Phage φ3T has a different orientation of its *sprA* gene in the viral genome and integrates its prophage in the *kamA* gene, coding for lysine 2,3-aminomutase [28]. Its *attL* and *attR* sites have a 5-bp conserved core (CCTAC) that is likely to represent the DNA breakpoint for integration. Adjacent to this core sequence, there are imperfect inverted repeat sequences (23-24 bp long) that presumably provide binding sites for a site-specific recombinase [55]. Thus, the φ3T integrase system differs from that of SPβ. This may explain the possibility of φ3T-SPβ double lysogeny, despite the significant similarity between the two phages [29]. Phage H2 lysogenises *B.* *amyloliquefaciens* H [CP041693.1] by integrating between two hypothetical genes. However, it also lysogenises *B.* *subtilis* and integrates itself between the *tyrA* and *metB* loci [38]. A comparison of the H2 core *attP* site with the relevant region of the *B.* *subtilis* genome [NC_000964.3] revealed only a putative imperfect *attB* sequence (5'-CCCttTAaAAATAACTA-3') at positions 2,333,200-2,333,216. The recent SPβ-like isolates Goe12 [MT601273.1] and Goe13 [MT601274.1] have the same core *attP* site as SPβ [52] and probably have the same integration locus as SPβ. In contrast, the *sprA* orientation in Goe11 [MT601272.1] resembles that found in φ3T. Analysis of a Goe11 lysogen revealed a putative integration site identical to that of φ3T (unpublished personal data). Integration site data of SPβ-related isolates are presented in Table 2.

**Table 2:** Known core *attP* sites of SPβ-related phages

Phage strain	Host	*attP* sequence (5′– > 3′)	*attB* sequence (5′– > 3′)	Integration locus
SPβ	*B. subtilis* 168	**ACAG**AT*AA*/AG**CTGT**AT	**ACAG**AT*AA*/AG**CTGT**AT	*spsM*
H2	*B. amyloliquefaciens* H	CCCTATAAATAACTA	CCCTATAAATAACTA	Intergenic
H2	*B. subtilis* 168	CCCTATAAATAACTA*	CCCttTAaAAATAACTA*	Intergenic*
φ3T	*B. subtilis* 168	aaaatgacataCCTACtgtgttttta	gctatgcggttCCTACctttgtcgtt	*kamA*
Goe11	*B. subtilis* ∆6	aaaatgacataCCTACtgtgtttttt*	gctatgcggttCCTACctttgtcgtt*	kamA*
Goe12	*B. subtilis* ∆6	**ACAG**AT*AA*/AG**CTGT**AT*	**ACAG**AT*AA*/AG**CTGT**AT*	spsM*
Goe13	*B. subtilis* ∆6	**ACAG**AT*AA*/AG**CTGT**AT*	**ACAG**AT*AA*/AG**CTGT**AT*	spsM*

The SPβ *att* sites were oriented based on Abe et al., 2017 [52]. Capital letters represent the inverted repeat recognised by SprA, a forward slash indicates the cleavage site, and underlined italic letters represent the 3' overhangs. The *att* sites of φ3T are presented as described by Suzuki et al., 2020 [55]. Capital letters indicate the conserved core, and lowercase letters indicate the associated imperfect repeat sequences. The *att* site sequences and integration loci marked with a star (*) are based on personal bioinformatic investigations and have not been confirmed experimentally.

The regulation of the integration process has not yet been investigated. The large serine recombinase SprA is expressed continuously and is responsible for integrating the phage genome into the bacterial chromosome [53], but the prophage itself is established just after the decision by the arbitrium system [56], implying a further layer of regulation.

Not much is known about how SPβ-like phages maintain their lysogenic state. The ~22-kDa d protein of SPβ [GenBank no. M13821.1], when ectopically expressed in an SPβ-sensitive host, prevents lytic replication or lysogenisation of the bacterium by SPβ. This protein seems to be constitutively synthesised, but its production is most likely regulated by other phage- encoded factors. It is either unstable or has an elevated turnover rate *in vivo*. It has been successfully expressed in a *B.* *subtilis* SPβ c2-free background, but it could not be detected via immunoblotting methods in an SPβ c2 lysogen [45]. However, the d protein is functional and biologically active in SPβ c1 and c2. It is neither responsible for the clear-plaque phenotype of SPβ c1/c10 nor the temperature sensitivity of c2, as those strains have been shown to have an intact d gene [45]. A frameshift mutation was engineered to inactivate the d protein. Introduction of the mutated d gene into SPβ-sensitive *B*. *subtilis* does not confer resistance to SPβ. When the damaged d gene is introduced into SPβ c1, it no longer has a clear-plaque phenotype but instead produces turbid plaques characteristic of lysogenic bacteria. Those lysogens are unstable and lose the prophage in further passages. In the temperature-sensitive SPβ c2 strain, the introduction of the mutated d gene leads to a clear-plaque phenotype, indicating the inability to form lysogens [45]. Thus, inactivation of the d protein in the clear-plaque mutant c1 and the temperature-sensitive-mutant c2 results in opposite phenotypes. This is intriguing, since complementation experiments suggest that both phenotypes are attributable to the same gene [85]. These observations point to a complex process of lysogeny establishment and maintenance in SPβ-related phages with potentially yet unknown components. Some, for example, may be located between *sprA* (*yokA*) and *sunI* (*yolF*), as a deletion of this region in the SPβ c2 del3 mutant has been shown to be associated with unstable lysogeny [63].

## Host conversion

Lysogenic conversion is the alteration of the properties of the host by its prophage, which introduces new genetic information into the bacterial chromosome. As mentioned above, this phenomenon is sometimes associated with the transformation of harmless bacteria into pathogens [86]. It is unlikely that SPβ-related phages turn their hosts into pathogens, as *B.* *subtilis* is generally recognised as safe and is even used for food production [87]. However, SPβ carries a gene (*yokG*) encoding a protein similar to the insecticidal delta endotoxin of *B.* *thuringiensis*, but its biological role remains unclear [44].

Regarding nucleotide metabolism, φ3T can convert its host from deoxyribosylthymine auxotrophy to prototrophy through the phage-encoded thymidylate synthase gene *thyP3* [5, 6]. SPβ does not have a thymidylate synthase gene, while its original host *B.* *subtilis* 168 contains two genes for this function (*thyA* -BSU_17680 and *thyB* - BSU_21820) [14, 15]. Still, SPβ has the ability to acquire the *thyP3* gene from φ3T through phage-phage homologous recombination during a mixed infection, or through the transformation of *B.* *subtilis* 168 with the corresponding φ3T DNA fragment [88, 89].

The *sspC* gene, provided by SPβ [44], encodes an α/β-type small acid-soluble spore protein (SASP) [90, 91], which stabilises spore DNA and increases spore resistance to UV light [92]. Its transcription is regulated by the forespore-specific SigG factor and is thus part of the host sporulation regulatory network [93].

SPβ contains the genes *sunI* (*yolF*), *sunA* (*yolG*), *sunT* (*yolH*), *bhdA* (*yolI*), *sunS* (*yolU*), and *bdbB* (*yolK*), with which it can weaponise the host with sublancin 168 [94, 95]. The *sunA* gene codes for the pre-peptide [96] which is post-translationally glycosylated by SunS at its cysteine residue 22 [97]. The remaining four cysteines are oxidised by BdbA and, in particular, by BdbB, resulting in two disulfide bounds [98, 99]. SunT exports this glycopeptide by proteolytic cleavage of the leader peptide [98]. The exported sublancin 168 inhibits the growth of many Gram-positive bacteria [100]. The active form of sublancin 168 is imported into sensitive cells by a glucose phosphate transferase system and inhibits synthesis of DNA, RNA, and protein [101]. SunI provides immunity against sublancin 168 and thus protects the SPβ lysogen [102]. While the *sunA* gene is regulated, *sunI* is constitutively expressed [54, 102]. The differential expression of the *sunA* gene in specific cell types during the late exponential and stationary growth phase enables *B. subtilis* to compete for its resources [103].

The SPβ *yokD* gene codes for a putative aminoglycoside N^3^'-acetyltransferase. This typically bacterial enzyme can confer resistance to aminoglycoside antibiotics, such as gentamicin or kanamycin [104, 105]. However, it remains unclear whether YokD plays a particular role in SPβ biology.

The SPβ *nonA* gene endows its host with the capability of protecting itself against unrelated lytic phages such as SP10 by aborting their infections. The small transmembrane protein NonA inhibits the synthesis of the capsid protein of SP10 and is regulated by sigma factors encoded by SP10 itself (Orf199-Orf200) [3, 4].

## A selfish phage

The production of sublancin 168 by SPβ may benefit the host but may also turn against it. During periods of adequate nutrient supply and logarithmic growth, *B.* *subtilis* frequently loses the SPβ prophage [50]. When entering the stationary phase, the remaining lysogens activate the sublancin 168 production and thereby eliminate all SPβ-free cells [103]. This reveals that sublancin 168 is also part of the prophage maintenance system.

An even more selfish aspect of SPβ can be found in its three toxin-antitoxin (TA) systems. Such two-component systems consist of a stable toxin, which kills the cell or causes growth stasis if produced in a certain amount, and an unstable antitoxin that controls the toxin. TA systems were first discovered to be encoded by plasmids and were shown to contribute to their maintenance by killing the host if the plasmid got lost [106]. In such a case, the antitoxin levels decrease rapidly, allowing the stable toxin to kill the plasmid-free cell (reviewed in reference 107). The TA systems of SPβ may have a similar function.

The *bsrG*/SR4 system is a type I TA system consisting of an antisense ncRNA (SR4), which controls a toxin (BsrG) mRNA [108]. SR4 acts through RNA-RNA interaction by inhibiting the translation of *bsrG* and promoting its degradation [109]. The *yonT/yoyJ*/SR6 system is the second type I toxin-antitoxin (TA) system. The toxin genes *yonT* and *yoyJ* act independently but are transcribed on one polycistronic mRNA. The antisense ncRNA SR6 resides on the DNA strand opposite to that of the toxins genes and partially covers the coding region of both toxins. It interacts with the 3' untranslated region of *yonT* mRNA, thereby promoting its degradation by RNase III, and controls the translation of *yoyJ* by directly binding to its ribosome-binding site [110]. The only type II TA system identified in SPβ consists of YokI and YokJ proteins [111, 112]. Further investigation is needed to elucidate how those TAs are implemented in the regulatory circuits of SPβ and how they fulfil their biological function.

## Concluding remarks

Despite half a century of research on SPβ and the fact that its host, *B. subtilis* 168, is one of the best-studied prokaryotic model systems, there are still blind spots in our understanding of its biology. We barely know how SPβ maintains its prophage status and how it decides to enter the lytic cycle. Not much more is known about the replication of its genome and the assembly of the infectious virion. For the majority of genes, no function has been assigned. It is also not known which genes are essential for lytic or lysogenic replication. A plethora of open questions remain to be answered to understand the biology of phage SPβ.
